# Extinction drives a discontinuous temporal pattern of species–area relationships in a microbial microcosm system

**DOI:** 10.1038/s41598-023-30685-x

**Published:** 2023-03-06

**Authors:** Wei Deng, Na Li, Chao-Zhi Zhang, Rui An, Xiao-Yan Yang, Wen Xiao

**Affiliations:** 1 Institute of Eastern-Himalaya Biodiversity Research, Dali University, Dali, Yunnan China; 2 Collaborative Innovation Center for Biodiversity and Conservation in the Three Parallel Rivers Region of China, Dali, Yunnan China; 3 The Provincial Innovation Team of Biodiversity Conservation and Utility of the Three Parallel Rivers Region, Dali University, Dali, Yunnan China; 4 International Centre of Biodiversity and Primates Conservation, Dali, Yunnan China; 5 Center for Cultural Ecology in Northwest Yunnan, Dali, Yunnan China

**Keywords:** Ecology, Biogeography, Microbial ecology

## Abstract

As the most potential ecological "law", the mechanism of the species-area relationship (SAR) remains controversial. Essentially, the SAR addresses the relationship between regional area and biodiversity, shaped by speciation, extinction and dispersal processes. Extinction is the process of loss and a direct cause of species richness differences in community. Therefore, it is crucial to elucidate the role of extinction in shaping SAR. Since the extinction process has temporal dynamics, we propose the hypothesis that the occurrence of SAR should also have temporal dynamics. Here, we designed independent closed microcosm systems, in which dispersal/speciation can be excluded/neglected to reveal the role of extinction in shaping the temporal dynamics pattern of SAR. We find that extinction can shape SAR in this system independent of the dispersal and speciation process. Due to the temporal dynamics of the extinction, SAR was temporally discontinuous. The small-scale extinctions modified community structure to promote ecosystem stability and shaped SAR, while mass extinction pushed the microcosm system into the next successional stage and dismissed SAR. Our result suggested that SAR could serve as an indicator of ecosystem stability; moreover, temporal discontinuity can explain many controversies in SAR studies.

## Introduction

The species–area relationship (SAR) is the most studied and well-documented biodiversity pattern and is considered one of the few "laws" in ecology^1–4^. The SAR describes the positive correlation between species richness and habitat area, which has been widely used in biodiversity prediction and species extinction assessment and has played a fundamental role in understanding biodiversity patterns, species conservation planning and even the construction of biodiversity theory^5–8^. There are currently three dominant hypotheses regarding the formation of SARs: (1) decrease in area leads to an increase in extinction (the area per se hypothesis)^9–12^, (2) the increase in habitat heterogeneity due to the increase in area (the habitat diversity hypothesis)^13,14^, and (3) the increase in the number of individuals observed as the area increases (the passive sampling hypothesis)^15^. In addition, edge effects and resource concentration are also thought to have played a key role in the formation of SARs^16^. Of course, some researchers believe that the SAR results from a combination of the above effects^17^. However, to date, the relative contributions and exact operation mode of many SAR hypotheses have not been determined^18^. Thus, the mechanism underlying SAR formation still needs to be clarified.

Connor and McCoy argue that determining the mechanism of the SAR requires direct validation of a hypothesis through experimental design and exclusion of other factors^17^. For example, to conclude that habitat diversity is the only mechanism underlying a SAR, it is necessary not only to directly prove the effect of habitat diversity on species richness but also to prove that there is no relationship between extinction rate and area. Essentially, the SAR addresses the relationship between regional area and biodiversity, which is shaped by processes of speciation, extinction and dispersal. Therefore, studies of SAR formation mechanisms should use controlled experiments to explore the independent mechanisms of action of the three critical processes of speciation, dispersal, and extinction one by one. Along this line, we have successfully revealed the role of dispersal in shaping SAR and confirmed the passive sampling hypothesis^19,20^. These research examples confirm the feasibility of the above research idea and are expected to elucidate the mechanism of SAR formation further.

A re-examination of the hypotheses of SAR formation mechanisms reveals that, except for the passive sampling hypothesis, each hypothesis of SAR includes the role of extinction in shaping SAR. The widely accepted area per se hypothesis in particular emphasizes the effect of extinction. The habitat diversity hypothesis also implicitly assumes that with increased habitat diversity, extinction rates will decrease. Therefore, to clarify the formation mechanism of the SAR, extinction should be a research focus. To determine the independent mechanism of extinction, an experimental design that excludes many influencing factors, such as dispersal, habitat diversity, resource availability, biodiversity formation background^21^, and sampling effects, is needed.

This study constructed an independently sealed microcosm system with homogeneously mixed Chinese pao cai (fermented vegetables) soup and opaque closed culture flasks. The use of amplicon sequencing methods to conduct a comprehensive assessment of microbial diversity in the microcosm. In this system, the historical background of microbial diversity is consistent, the environment is homogeneous, resource density is consistent, and the impacts of dispersal are completely avoided. The speciation process in the 60-day experimental system can be negligible due to the short experimental period, meaning that extinction should be the dominant force shaping the biodiversity of the microcosm system. Studies have found that extinction is a dynamic process^22,23^. Therefore, we monitored the biodiversity of microcosm systems at 26 time points over a 60-day period. We hypothesised the following: (1) Extinction would be the primary mechanism of SAR in microcosm systems; (2) extinction in the microcosm would be temporally dynamic, with mass extinction promoting the succession of microcosmic ecosystems; and (3) SAR occurrence would be temporally dynamic, consistent with the community's successional phase.

## Materials and methods

### Preparation of the pao cai soup

First, 35 kg of white radish (*Raphanus sativus*), 35 kg of cabbage (*Brassica oleracea*), 2 kg of chili pepper (*Capsicum frutescens*), 1 kg of ginger (*Zingiber officinale*), 1 kg of peppercorns (*Zanthoxylum bungeanum*), 2.5 kg of rock sugar, and 210 kg of cold boiled water (containing 6% salt) were divided into six ceramic jars. After 7 days of natural fermentation at room temperature, the pao cai was filtered out with sterile gauze to obtain 200 kg of pao cai soup. To ensure an even distribution of microorganisms in the soup, the soup was mixed well and then left to rest for 12 h, the supernatant was taken, and the soup was left to rest for 12 h again.

The plants used in this study were cultivated vegetables which purchased from the vegetable market at the study site. All local, national or international guidelines and legislation were adhered to in the production of this study.

### Establishment of the microcosm system

**Figure 1:**
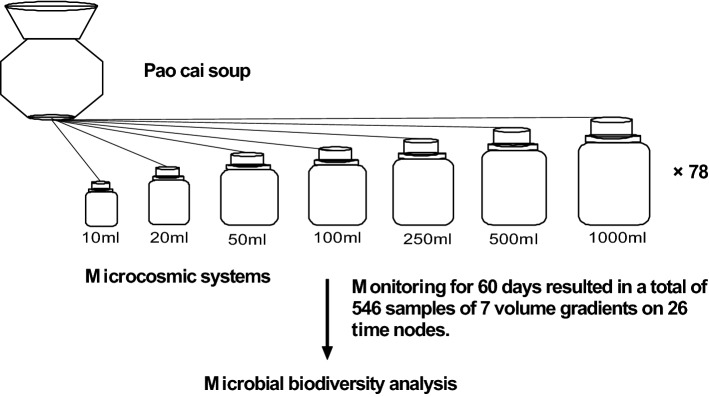
Schematic diagram of the establishment of the microcosmic system.

Seventy-eight for each size of 10 ml, 20 ml, 50 ml, 100 ml, 250 ml, 500 ml, and 1000 ml sterile glass culture flasks were filled with pao cai soup, the bottle mouth was sealed with sterile sealing film, and the bottle was capped without leaving any air (Fig. 1). Each flask became a microcosm and was cultured in a 25 °C incubator.

### Sample collection

Before the microcosm system was established, a sample of well-mixed pao cai soup was taken as a reference to establish background biodiversity. The microbial community dynamics should change the fastest at the beginning of the microcosm system establishment and gradually become slower over time. Considering the workload and cost, this study collected samples daily for 1–10 day after the establishment of the microcosm and then collected every 2 days for 10–30 day and every 5 days for 30–60 day. Three different microcosms of the same volume were established. Monitoring was carried out for 60 days, and a total of 546 samples of 7 volumetric gradients were obtained at 26 time points. At the time of sampling, the pao cai soup in the microcosm was mixed, and 50 mL of sample (10 mL of sample was collected for microcosm systems with a volume of less than 50 mL) was collected. The sample was centrifuged at 8000 rpm for 10 min, the supernatant was collected for pH determination, and the pellet was stored in a − 80 °C freezer.

### Microbial analyses

Microbial DNA was extracted from pao cai samples using the E.Z.N.A.^®^ Soil DNA Kit (Omega Biotek, Norcross, GA, U.S.) according to the manufacturer’s protocols. For bacteria, we targeted the V3-V4 region of the 16S ribosomal RNA (rRNA) gene, using the 338F (5′-ACTCCTACGGGAGGCAGCAG-3′) and 806R (5′-GGACTACHVGGGTWTCTAAT-3′) primer pairs. For fungi, we targeted the ITS1-1F region of the nuclear ribosomal internal transcribed spacer region (ITS rDNA) gene, using ITS1-1F-F (5′-CTTGGTCATTTAGAGGAAGTAA-3′) and ITS-1F-R (5′-GCTGCGTTCTTCATCGATGC-3′). PCRs were performed in triplicate in a 20 μL mixture containing 4 μL of 5 × FastPfu Buffer, 2 μL of 2.5 mM dNTPs, 0.8 μL of each primer (5 μM), 0.4 μL of FastPfu Polymerase and 10 ng of template DNA. The PCR program for the 16S rRNA gene was as follows: 3 min of denaturation at 95 °C; 27 cycles of 30 s at 95 °C, 30 s of annealing at 55 °C, and 45 s of elongation at 72 °C; and a final extension at 72 °C for 10 min. For the ITS1-1F region, the PCR program was as follows: samples were initially denatured at 98 °C for 1 min, followed by 30 cycles of denaturation at 98 °C for 10 s, primer annealing at 50 °C for 30 s, and extension at 72 °C for 30 s. A final extension step of 5 min at 72 °C was added to ensure complete amplification of the target region. The resulting PCR products were extracted from a 2% agarose gel, further purified using the AxyPrep DNA Gel Extraction Kit (Axygen Biosciences, Union City, CA, USA) and quantified using QuantiFluor™-ST (Promega, Madison, WI, USA).

Purified amplicons were pooled in equimolar amounts and paired-end sequenced (2 × 300) on an Illumina NovaSeq platform (Illumina, San Diego, CA, USA) according to standard protocols. The analysis was conducted by following the "Atacama soil microbiome tutorial" of QIIME2 docs along with customized program scripts (https://docs.qiime2.org/2019.1/). Briefly, raw data FASTQ files were imported in the QIIME2 system using the qiime tools import program. Demultiplexed sequences from each sample were quality filtered, trimmed, denoised, and merged, and then the chimeric sequences were identified and removed using the QIIME2 DADA2 plugin to obtain the feature table of amplicon sequence variants (ASVs)^24^. Compared with traditional OTU that clusters at 97% similarity, ASV has higher accuracy, equivalent to 99% similarity clustering. The QIIME2 feature-classifier plugin was then used to align ASV sequences to the pretrained GREENGENES 13_8 99% database (trimmed to the V3-V4 region bound by the 338F/806R primer pair for bacteria) and UNITE database (for fungi) to generate the taxonomy table^25^. Any contaminating mitochondrial and chloroplast sequences were filtered using the QIIME2 feature-table plugin. Based on the sequence number of the lowest sample, perform the resampling to make the sequence number equal for each sample. Due to the random nature of sequencing, ASVs specific to each sample in this study were present. To reduce the uncertainty introduced by the sequencing process, we filtered out rare ASVs with less than 0.001% of the total sequence volume.

### Data analysis

In this study, the data of fungi and bacteria were integrated and analyzed, and all microbial diversity appearing in the text represent the sum of all fungi and bacteria. Species richness is equal to the number of taxa, which is equal to the total number of all bacterial and fungal ASVs. The vegan package in R 4.2.1 was used to calculate the species richness of each sample based on the ASV feature table^26^. Using flask volume instead of area, SAR fitting was performed using a semi-logarithmic model, and its significance was tested. The semi-logarithmic model is the function S = c + b*logA, where S is species richness, A is area (in this case, volume is used instead), and b and c are fit parameters^27^.

The microcosmic system in this study is hermetically sealed, and all microorganisms originate from a single portion of well-mixed paocai soup (ie species pool). The speciation process in the 60-day experimental system should be negligible due to the short experimental period. The extinction rate of a microcosm system is equal to the number of ASVs lost in the microcosm system compared to the species pool divided by the total number of ASVs in the species pool. The extinction rate is the number of extinct ASVs in each system compared to the species pool. Pearson correlation analysis was performed with volume as the independent variable and extinction rate as the dependent variable to determine the correlation between volume and extinction rate at each time point. When microorganisms of a microcosmic system disappear entirely or cannot be detected, the microcosm is recorded as an annihilated microcosm. The annihilation rate at a time point is equal to the number of microcosms annihilated at that time, divided by the total number of microcosms. The difference between the extinction rate and annihilation rate defined in this paper is that the extinction rate is for ASVs within each sample, and the annihilation rate is for microcosmic system at each sampling time point. The two indicators jointly characterize the local extinction of microorganisms from different perspectives. Non-linear regression with a bell-shaped form was performed with time as an independent variable and pH and annihilation rate as dependent variables, and regression lines were plotted based on R 4.2.1.

According to the taxonomy table, bacterial ASVs were divided into acid-producing and non-acid-producing categories, and their extinction rates were calculated separately. The agricola, ggplot2, vegan and ggpubr packages were used to draw alpha diversity box plots and perform the Wilcoxon rank sum test for differences between groups^26,28–30^. Non-metric multidimensional scaling (NMDS) analysis was performed with the vegan package based on Bray–Curtis dissimilarity. In addition, the potential Kyoto Encyclopedia of Genes and Genomes (KEGG) orthologue (KO) functional profiles of microbial communities were predicted with PICRUSt^31^. Resistance-related genes were screened using the gene function predictions. The relationship between the relative abundance of resistance-related genes and the volume of the microcosm was analysed by Pearson correlation, and a forest map was plotted to present the results.

## Results

A total of 19,637,820 and 6,553,883 high-quality sequences were obtained for bacteria and fungi across all samples, respectively, with an average of 37,838 ± 4849 (bacteria, mean ± SD) and 18,942 ± 17,186 (fungi, mean ± SD) sequences detected per sample. After removal of rare ASVs, we obtained 783 and 889 ASVs for bacteria and fungi, respectively.

### The temporal dynamics of SARs

**Figure 2:**
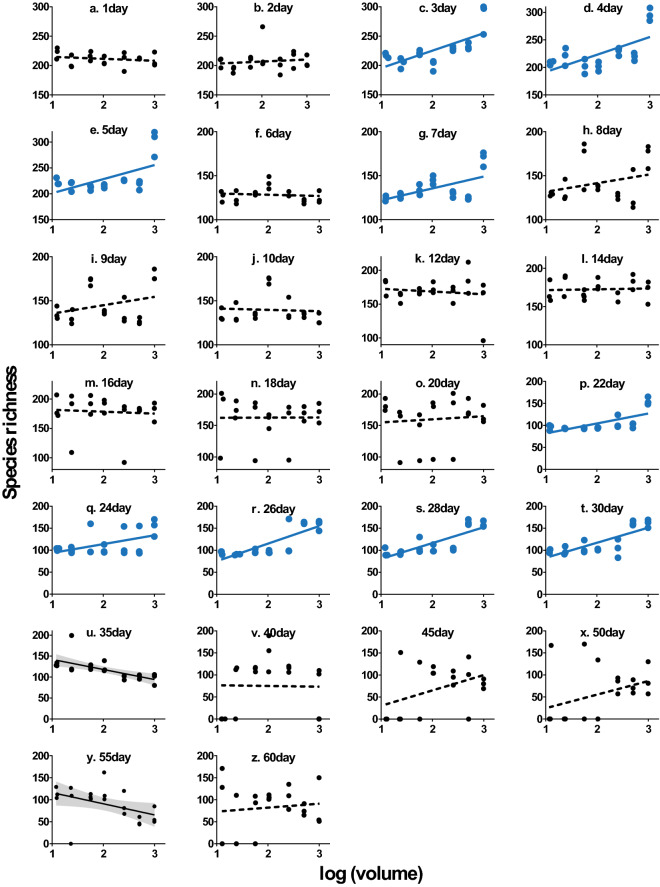
Microbial SAR at 60 days in the microcosm system. The regression line is from a linear model (volume is normally converted to the log scale). A solid line indicates a significant correlation, and a dashed line indicates that the correlation is not significant. A blue band represents the 95% confidence interval of a positive SAR, and a grey band represents the 95% confidence interval of a negative SAR (see Supplementary Table 1 for linear regression results).

During the 60 days of monitoring, a significant SAR occurred in the microcosm system in only 3–5 day, day 7, and 22–30 day (Fig. 2c,d,e,g,p,q,r,s,t; Supplementary Table 1). At 35 and 55 day, species richness in the microcosm system decreased significantly with increasing volume (Fig. 2u,y). At the remaining time points, there was no SAR in the microbial community in the closed system (Fig. 2a,b,f,h,i,j,k,l,m,n,o,v,w,x,z).

### Extinction drove the microbial SAR in the microcosm system

**Figure 3:**
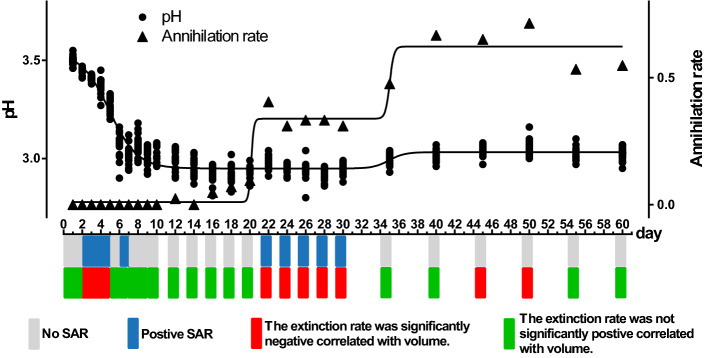
Sixty-day pH values and annihilation rates of the microcosm system, the SAR at each time point, and the relationship between extinction rate and volume. The regression line is from the model with a bell-shaped form (pH: p = 0.048, R^2^ = 0.927; annihilation rate: p = 0.045, R^2^ = 0.976). A grey block indicates that there is no microbial SAR at the corresponding time point, while a blue block indicates that there is. A green block indicates that the extinction rate is not negatively correlated with volume, while a red block indicates that the extinction rate is significantly negatively correlated with volume (see Supplementary Table 2 for statistical test results). To remove the effect of inconsistent sampling efforts, the above data were presented at intervals of 5 days, and the results showed that the above phase changes did not change (see Supplementary Fig. 1).

Except on day 7, the extinction rates of ASVs during the periods with a SAR were significantly inversely correlated with the volume of the microcosm (Fig. 3, Supplementary Table 2).

### Phased succession of the microcosm system

The pH in the microcosm system showed a phased change. Specifically, it decreased rapidly on 1–10 day, remained stable on 12–35 day, and recovered on 40–60 day. Accordingly, the microcosm system can be divided into three phases: Phase 1 (1–10 day), Phase 2 (12–40 day), and Phase 3 (45–60 day).

**Figure 4:**
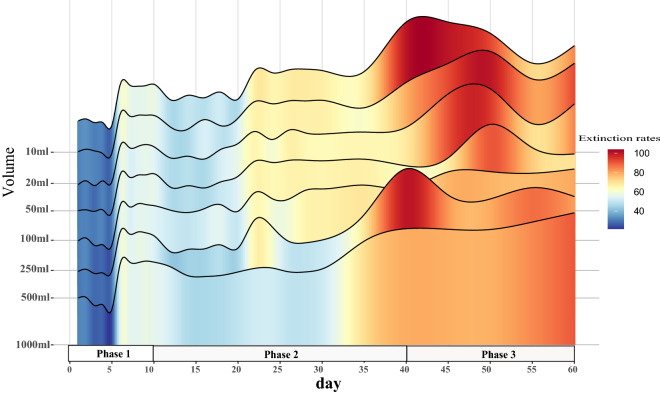
Dynamic change in the extinction rates of microcosm systems. Colours from blue to red represent a low to high extinction rate.

In line with this finding, annihilation of the microcosm system did not occur in Phase 1, and there was systematic annihilation in Phase 2, with the annihilation rate exceeding 50% in Phase 3. The extinction rate at each volume of the microcosm system showed a temporal dynamic change pattern. The extinction rate increased significantly in the system on day 6 and day 40, and a mass extinction event occurred. The average extinction rate per unit volume in Phase 1 was 43.24% ± 13.07, the average extinction rate per unit volume in Phase 2 was 56.59% ± 8.25, and the average extinction rate per unit volume in Phase 3 was 82.42% ± 10.24 (Fig. 4).

**Figure 5:**
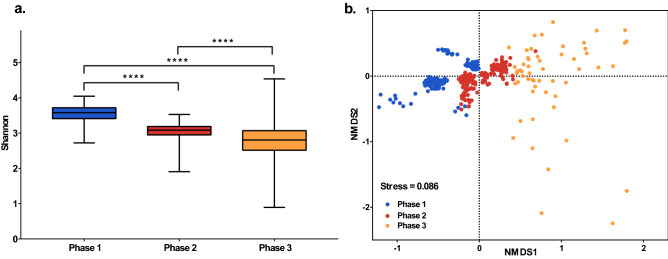
(**a**) Differences in alpha diversity based on the Shannon index (Wilcoxon rank sum test, p < 0.0001). (**b**) NMDS analysis based on Bray–Curtis dissimilarity, showing differentiation in Phases 1–3 (stress = 0.0086).

Corresponding to the three phases of the microcosm system, the alpha diversity and beta diversity of the microbial community also exhibited phased differentiation. The Shannon diversity index differed significantly over the three phases (p < 0.0001, Fig. 5a). Non-metric multidimensional scaling (NMDS) analysis showed that the microbial communities exhibited phased differentiation over the three phases (stress = 0.087, Fig. 5b).

### Phased changes in the functional traits of microcosm systems

**Figure 6:**
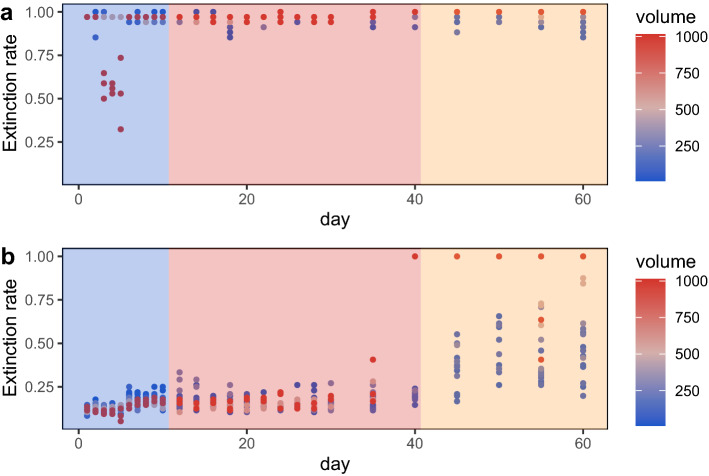
(**a**) Extinction rate of non-acid-producing bacteria. (**b**) Extinction rate of acid-producing bacteria. The colour depth of the dots represents the volume of the microcosm system, increasing in volume from blue to red. The blue background represents Phase 1, red represents Phase 2, and yellow represents Phase 3.

The ASVs of non-acid-producing bacteria at each volume rapidly went extinct. The extinction rate was low in the 1000 mL microcosm system only at 3–5 day (Fig. 6a). Acid-producing bacterial ASVs did not fluctuate significantly before 35 days but gradually increased in Phase 3 (Fig. 6b).

Functional gene prediction revealed two genes related to antibiotic drug resistance, two anti-metabolic bacteriostatic drug resistance genes, and two anti-cancer drug resistance genes. Taking beta lactam resistance, anti-folate resistance, and platinum drug resistance genes as examples, the relative abundance of the three types of genes showed different correlation trends with the volume of the microcosm system at different times. Beta lactam resistance and anti-folate resistance genes had a negative correlation with microcosm volume on 3–5 day of Phase 1 and day 22 and 24 of Phase C, which also showed a SAR (Supplementary Fig. 2). On 22–30 day, when Phase 2 showed a SAR, the relative abundance of the platinum drug resistance gene tended to be negatively correlated with the volume of the microcosm (Supplementary Fig. 2).

## Discussion

This study finds that the occurrence of a SAR is discontinuous over time. The microcosm system had a SAR on days 3–5, 7, and 22–30, accounting for 34.61% of the observation time points and 21.67% of the entire observation period (Fig. 2). The SAR could be considered as the ecological "law" to some extent, generally ignores the time what is one of the three key elements (time, space and scale) of the ecological research. In recent years, researchers have proposed the species-time-area relationship (STAR) to perfect the SAR, taking into account the temporal dynamics of species richness^32^. However, the STAR still assumes that a SAR will exist continuously in a time series.

When a SAR can be observed in microcosm systems, the extinction rate and the system's volume negatively correlate. This shows that extinction was the primary mechanism driving the SAR in the closed microcosm, which is entirely consistent with our hypothesis. Surprisingly, extinction also led to the disappearance of the SAR. The mass extinctions on day 6 and day 40 wiped out previously stable SARs (the extinction rate on day 6 increased by 27.43% from that on the previous day, and the extinction rate on day 40 increased by 12.50% from that on day 35) (Figs. 3, 4). The timing of these two mass extinctions coincided with the change in the pH of the microcosm environment. The process by which a community change with the environment has a strong consensus in ecology. This study observed the coupling of ecological community and their environments. No doubt that acid-producing taxa in the pao cai microbial community drove the decline in environmental pH^33^. In turn, the alpha and beta diversity of the microbial communities presented three phases divided by shifts in pH (Fig. 5). Arguably, antagonism, or civil war between microbes (i.e. competition), led to extinction throughout the experimental period and drove the succession of the microcosm ecosystems. In Phase 1, the acid-producing microbial taxa rapidly lowered the environmental pH, leading to the extinction of the non-acid-producing taxa (Fig. 6). The emergence of antibiotic drug resistance genes, anti-metabolic bacteriostatic drug resistance genes and anti-cancer drug resistance genes represents the generation of antagonistic metabolites in the microcosmic system and the potential for extinction of some taxa due to the mutual antagonism of microorganisms in the microcosmic system. The extinction caused by antagonistic metabolites was manifested throughout the experimental cycle. The relative abundance of various antagonistic metabolite suppressor genes appeared to be correlated with the planet volume at the time of SAR production, respectively (Supplementary Fig. 1). Each full successional stage should include an adjustment stage (early stage), a stabilization stage (middle stage) and a collapsing stage (terminal stage). In the early stages of each phase, the gradual occurrence of extinctions regulated the community, and in the middle stage, it stabilized the community. The SAR occurred during the stable stage of the community. As environmental changes continued to accumulate, a mass extinction occurred at the end of each phase, prompting community succession to the next stage and causing the SAR to disappear until the next stabilization stage. Phase 2 observed in this study should be considered such a complete successional stage.

In general, in our system, extinction shaped SARs but also destroys them; the SARs are discontinuous in time and exist only during stages of ecosystem stabilization. These findings are essential for solving the ecological problems related to SARs and can also be a good reference for sustainable development science. First, in microbes, the life cycle is short, community succession is fast, and community succession can be observed over a short amount of time^34^. Thus, in the future, microorganisms should become important taxa for explaining the SAR mechanism and community development. Second, SARs only occur in periods of relative stability, so the presence or absence of a SAR could serve as an indicator of ecosystem stability. We predict that a SAR will not exist during phases of rapid urbanization, cyanobacterial outbreaks, biological invasion, and other disruptions when biodiversity is plummeting, nor should a SAR exist in the history of major biodiversity explosions and mass extinctions. In the future, we should pay attention to managing extinction drivers within communities and enhance the research on the role of speciation and dispersal in maintaining the stability of ecosystems to achieve sustainable development on a long time scale.

## Supplementary Information

Supplementary Information.

## Data Availability

The raw sequence data reported in this paper have been deposited in the Genome Sequence Archive (Genomics, Proteomics & Bioinformatics 2021) in National Genomics Data Center (Nucleic Acids Res 2022), China National Center for Bioinformation/Beijing Institute of Genomics, Chinese Academy of Sciences (GSA: CRA008411) that are publicly accessible at https://bigd.big.ac.cn/gsa/browse/CRA008411.
