# Supplementary material for: Extinction drives a discontinuous temporal pattern of species–area relationships in a microbial microcosm system
Source: Sci Rep. 2023 Mar 6;13:3720. doi: 10.1038/s41598-023-30685-x (PMC9988864; doi:10.1038/s41598-023-30685-x)
Supplement: Supplementary file 1 — Supplementary Information. [file 41598_2023_30685_MOESM1_ESM.docx]

**Supplementary Information**

**
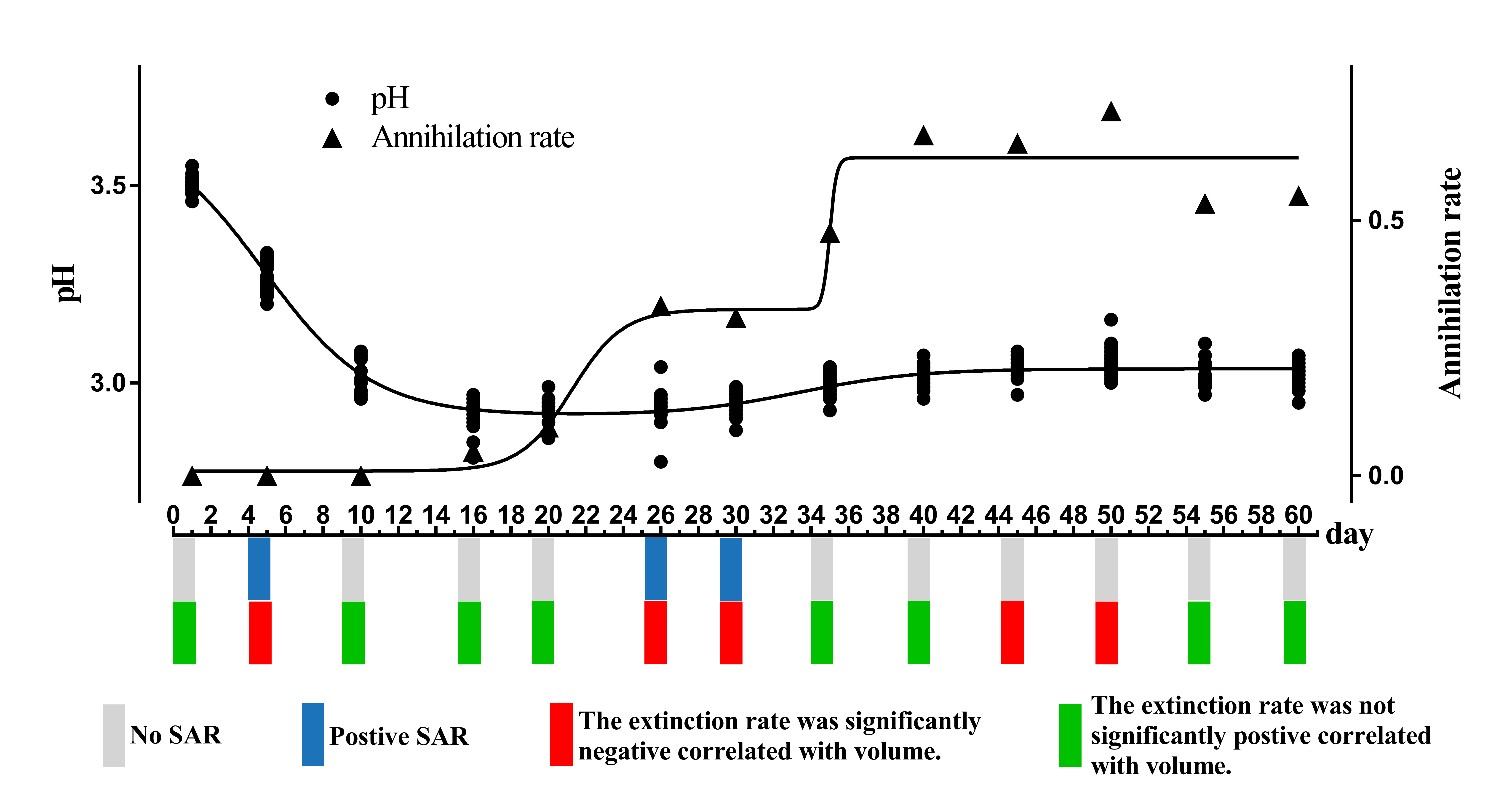
Supplementary Fig. 1** Sixty-day pH values and annihilation rates of the microcosm system, the SAR at each time point, and the relationship between extinction rate and volume. To remove the effect of inconsistent sampling efforts, the above data were presented at intervals of 5 days. The regression line is from the model with a bell-shaped form. A grey block indicates that there is no microbial SAR at the corresponding time point, while a blue block indicates that there is. A green block indicates that the extinction rate is not negatively correlated with volume, while a red block indicates that the extinction rate is significantly negatively correlated with volume.

**
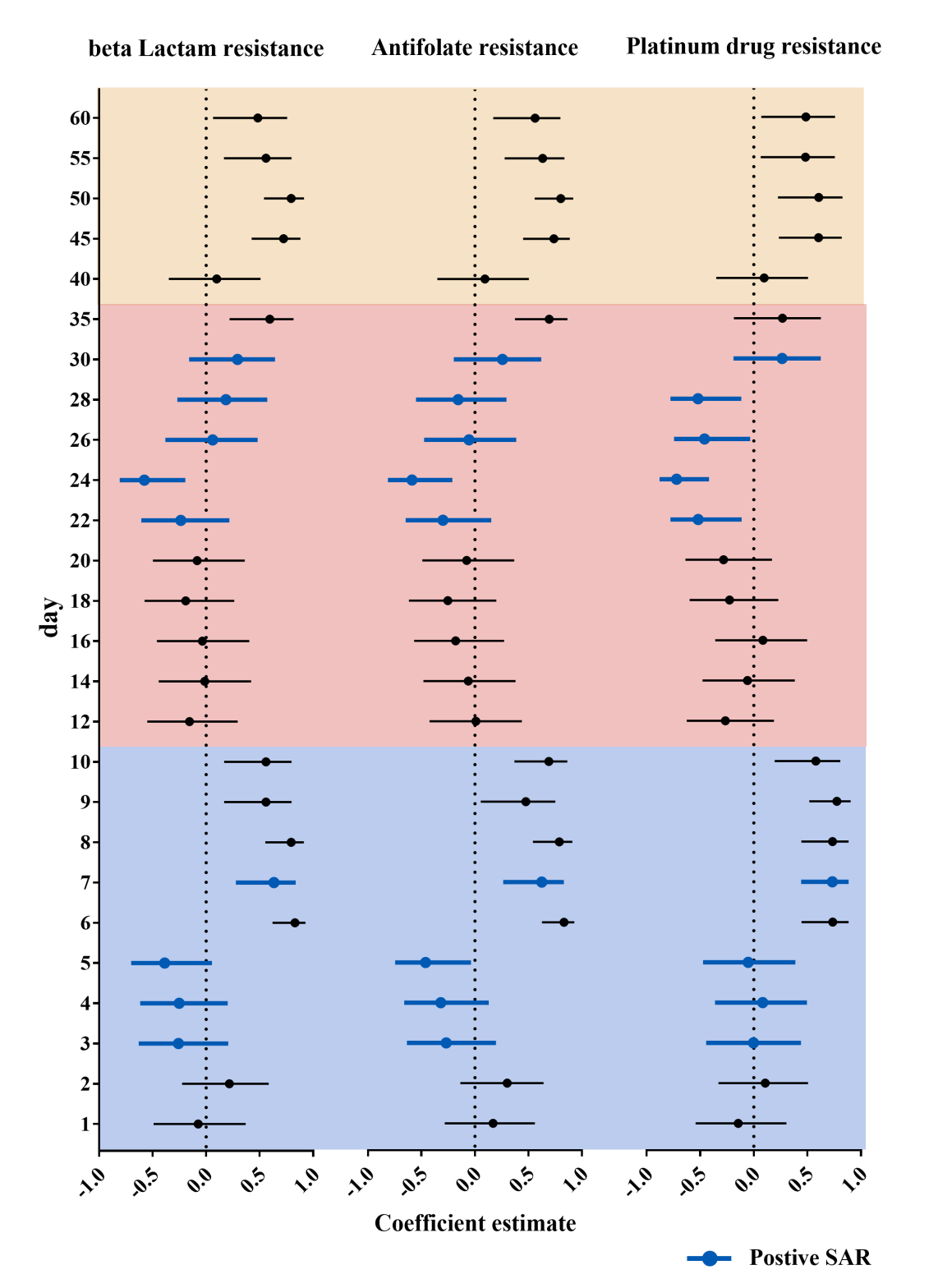
Supplementary Fig. 2 Correlation of the relative abundance of beta lactam resistance, antifolate resistance and platinum drug resistance genes with the volume of the microcosm systems.** Correlation coefficients and p values are shown in Table S3. The horizontal axis is the correlation coefficient, and the vertical axis is time. The right side of the dotted line indicates positively correlated, and the left side of the dashed line indicates negatively correlated. Blue corresponds to Phase 1, red corresponds to Phase 2, and yellow corresponds to Phase 3.

Supplementary Table 1 Statistical test results of microbial SAR curves

| day | R2 | p |  | day | R2 | p |
| --- | --- | --- | --- | --- | --- | --- |
| 1 | 0.0466 | 0.3477 |  | 18 | <0.0001 | 0.9810 |
| 2 | 0.0182 | 0.5604 |  | 20 | 0.0087 | 0.6868 |
| 3 | 0.4842 | 0.0005*** |  | 22 | 0.4580 | 0.0008*** |
| 4 | 0.4202 | 0.0015** |  | 24 | 0.2417 | 0.0236* |
| 5 | 0.3273 | 0.0067** |  | 26 | 0.6539 | <0.0001*** |
| 6 | 0.0139 | 0.6109 |  | 28 | 0.6477 | <0.0001*** |
| 7 | 0.3089 | 0.0089** |  | 30 | 0.6031 | <0.0001*** |
| 8 | 0.0817 | 0.2093 |  | 35 | 0.4391 | 0.0011** |
| 9 | 0.0916 | 0.1823 |  | 40 | <0.0001 | 0.9365 |
| 10 | 0.0040 | 0.7849 |  | 45 | 0.163 | 0.0775 |
| 12 | 0.0160 | 0.5847 |  | 50 | 0.1202 | 0.1237 |
| 14 | 0.0033 | 0.8053 |  | 55 | 0.2015 | 0.0412* |
| 16 | 0.0057 | 0.7451 |  | 60 | 0.01358 | 0.6150 |

* p < 0.05, ** p < 0.01, *** p < 0.001

Supplementary Table 2 Statistical test result of the correlation between extinction rate and volume

| day | r | p |  | day | r | p |
| --- | --- | --- | --- | --- | --- | --- |
| 1 | -0.1598 | 0.4889 |  | 18 | -0.1472 | 0.5244 |
| 2 | -0.3393 | 0.1324 |  | 20 | -0.1547 | 0.5032 |
| 3 | -0.6501 | 0.0014** |  | 22 | -0.6844 | 0.0006*** |
| 4 | -0.5659 | 0.0075** |  | 24 | -0.5127 | 0.0175* |
| 5 | -0.5152 | 0.0168* |  | 26 | -0.8193 | <0.0001*** |
| 6 | -0.3464 | 0.1240 |  | 28 | -0.8135 | <0.0001*** |
| 7 | -0.1967 | 0.3928 |  | 30 | -0.7717 | <0.0001*** |
| 8 | 0.03831 | 0.8691 |  | 35 | 0.5124 | 0.0176* |
| 9 | 0.00458 | 0.9843 |  | 40 | -0.0027 | 0.9906 |
| 10 | -0.3874 | 0.0827 |  | 45 | -0.5235 | 0.0149* |
| 12 | 0.1866 | 0.4180 |  | 50 | -0.6306 | 0.0022** |
| 14 | -0.2890 | 0.2039 |  | 55 | 0.4600 | 0.0359* |
| 16 | -0.1860 | 0.4195 |  | 60 | -0.0543 | 0.8151 |

* p < 0.05, ** p < 0.01, *** p < 0.001

Supplementary Table 3 Correlation between the relative abundance of beta Lactam resistance, Antifolate resistance and Platinum drug resistance genes and the volume of microsystems

|  | **Beta Lactam resistance** | |  | **Antifolate resistance** | |  | **Platinum drug resistance** | |
| --- | --- | --- | --- | --- | --- | --- | --- | --- |
| **day** | **r** | **p** |  | **r** | **p** |  | **r** | **p** |
| **1** | **-0.07477** | **0.7474** |  | **0.1705** | **0.4599** |  | **-0.1451** | **0.5302** |
| **2** | **0.2168** | **0.3325** |  | **0.3023** | **0.1716** |  | **0.1075** | **0.6338** |
| **3** | **-0.2592** | **0.2698** |  | **-0.2687** | **0.252** |  | **-0.00182** | **0.9939** |
| **4** | **-0.2523** | **0.2699** |  | **-0.3194** | **0.1581** |  | **0.08336** | **0.7194** |
| **5** | **-0.3872** | **0.0829** |  | **-0.4613** | **0.0353*** |  | **-0.05209** | **0.8226** |
| **6** | **0.8297** | **<0.0001****** |  | **0.8329** | **<0.0001****** |  | **0.7361** | **0.0001***** |
| **7** | **0.6333** | **0.0021**** |  | **0.625** | **0.0025**** |  | **0.7335** | **0.0002***** |
| **8** | **0.7946** | **<0.0001****** |  | **0.7892** | **<0.0001****** |  | **0.7354** | **0.0001***** |
| **9** | **0.5591** | **0.0084**** |  | **0.4755** | **0.0294*** |  | **0.7766** | **<0.0001****** |
| **10** | **0.5591** | **0.0084**** |  | **0.6907** | **0.0005***** |  | **0.5789** | **0.006**** |
| **12** | **-0.1564** | **0.4984** |  | **0.009825** | **0.9663** |  | **-0.2651** | **0.2455** |
| **14** | **-0.01384** | **0.9525** |  | **-0.06209** | **0.7892** |  | **-0.05839** | **0.9015** |
| **16** | **-0.03518** | **0.8797** |  | **-0.1794** | **0.4365** |  | **0.08576** | **0.7117** |
| **18** | **-0.1911** | **0.4067** |  | **-0.2527** | **0.2691** |  | **-0.2254** | **0.3258** |
| **20** | **-0.08462** | **0.7153** |  | **-0.07659** | **0.7414** |  | **-0.2822** | **0.2152** |
| **22** | **-0.2373** | **0.3004** |  | **-0.2984** | **0.1889** |  | **-0.5194** | **0.0158** |
| **24** | **-0.5779** | **0.0061**** |  | **-0.5887** | **0.005**** |  | **-0.7196** | **0.0002***** |
| **26** | **0.06125** | **0.792** |  | **-0.05442** | **0.8148** |  | **-0.4587** | **0.0365*** |
| **28** | **0.1839** | **0.4249** |  | **-0.1557** | **0.5002** |  | **-0.5209** | **0.0155*** |
| **30** | **0.2931** | **0.1972** |  | **0.2573** | **0.2622** |  | **0.264** | **0.2475** |
| **35** | **0.5941** | **0.0045**** |  | **0.6942** | **0.0005***** |  | **0.2674** | **0.2412** |
| **40** | **0.09809** | **0.6723** |  | **0.09461** | **0.6833** |  | **0.09696** | **0.6759** |
| **45** | **0.7239** | **0.0002***** |  | **0.7392** | **0.0001***** |  | **0.6054** | **0.0036**** |
| **50** | **0.7935** | **<0.0001****** |  | **0.8033** | **<0.0001****** |  | **0.6074** | **0.0045**** |
| **55** | **0.5581** | **0.0086**** |  | **0.6335** | **0.002**** |  | **0.483** | **0.0265*** |
| **60** | **0.483** | **0.0266*** |  | **0.5623** | **0.008**** |  | **0.4868** | **0.0252*** |
